# Small Cell Carcinoma of the Ovary, Pulmonary Type, With a Germline BRCA2 Mutation: A Report of a Rare Case

**DOI:** 10.7759/cureus.78894

**Published:** 2025-02-12

**Authors:** Asumi Misawa, Miyamoto Shingo, Tomoya Miyamura, Takafumi Ogawa, Miki Morioka

**Affiliations:** 1 Obstetrics and Gynecology, Showa University Fujigaoka Hospital, Yokohama, JPN; 2 Obstetrics and Gynecology, Showa University Northern Yokohama Hospital, Yokohama, JPN; 3 Pathology, Showa University Fujigaoka Hospital, Yokohama, JPN

**Keywords:** drug resistance, germline brca mutations, gynecologic oncology, gynec pathology, maintenance therapy, ovarian carcinoma, poly(adp-ribose) polymerase-1 (parp1) inhibitor, pulmonary type, rare cancers, small cell carcinoma of the ovary

## Abstract

Small cell carcinoma of the ovary, pulmonary type (SCCOPT), is a rare and aggressive malignancy with a poor prognosis. We report a rare case of this cancer in a patient with a germline *BRCA2* (g*BRCA2*)* *mutation. A 66-year-old female patient presented with abdominal distention, underwent a laparotomy, and was diagnosed with stage IVB SCCOPT. Histopathological examination revealed no other ovarian tumor components. Adjuvant platinum-based chemotherapy was administered, followed by maintenance therapy with poly(ADP-ribose) polymerase inhibitors (PARPi) because of the g*BRCA2* mutation. Despite expectations of a favorable outcome with the targeted maintenance therapy, disease progression was noted five months after starting maintenance therapy, and the patient died 12 months after surgery. The disease course suggests that maintenance therapy may have limited efficacy against this cancer, even in the presence of g*BRCA2* mutations. Further research is necessary to investigate the role of PARPi and establish more effective oncological treatment for this cancer.

## Introduction

Primary small cell carcinoma of the ovary is a rare and highly aggressive malignant neoplasm [1]. This cancer is histologically divided into two distinct subtypes that are considered different tumors: small cell carcinoma of the ovary, pulmonary type (SCCOPT), and small cell carcinoma of the ovary, hypercalcemic type (SCCOHT) [2].

SCCOPT typically affects women aged 50-60 years, whereas SCCOHT occurs in younger women [1]. Histopathologically, SCCOPT is similar to small cell carcinoma of the lung and is considered to be a neuroendocrine tumor [2]. In contrast, SCCOHT is characterized by *SMARCA4* mutations and is often associated with hypercalcemia [3].

Primary small cell carcinoma of the ovary accounts for fewer than 1% of ovarian cancers [1], and SCCOPT is even rarer [2]; the largest review of SCCOPT available includes only 38 patients [4]. Due to its rarity, genomic mutations in SCCOPT have not been thoroughly investigated, resulting in a paucity of data.

Although no established treatment guideline exists for SCCOPT, patients are typically managed with surgical resection followed by platinum-based chemotherapy. However, the prognosis remains extremely poor [4].

In common ovarian epithelial cancer, *BRCA* mutations, which impair homologous recombination repair, are well-studied and are associated with sensitivity to platinum-based chemotherapy and poly(ADP-ribose) polymerase inhibitors (PARPi) as maintenance therapy [5]. However, there have been no case reports of SCCOPT with germline *BRCA* (g*BRCA*) mutations nor any reports of patients with SCCOPT receiving PARPi as maintenance therapy.

We herein report a clinical case of SCCOPT, which consists solely of a small cell carcinoma component with a g*BRCA* mutation. To the best of our knowledge, this is the first case of its kind treated with surgery followed by platinum-based chemotherapy and PARPi maintenance therapy.

## Case presentation

**Figure 1:**
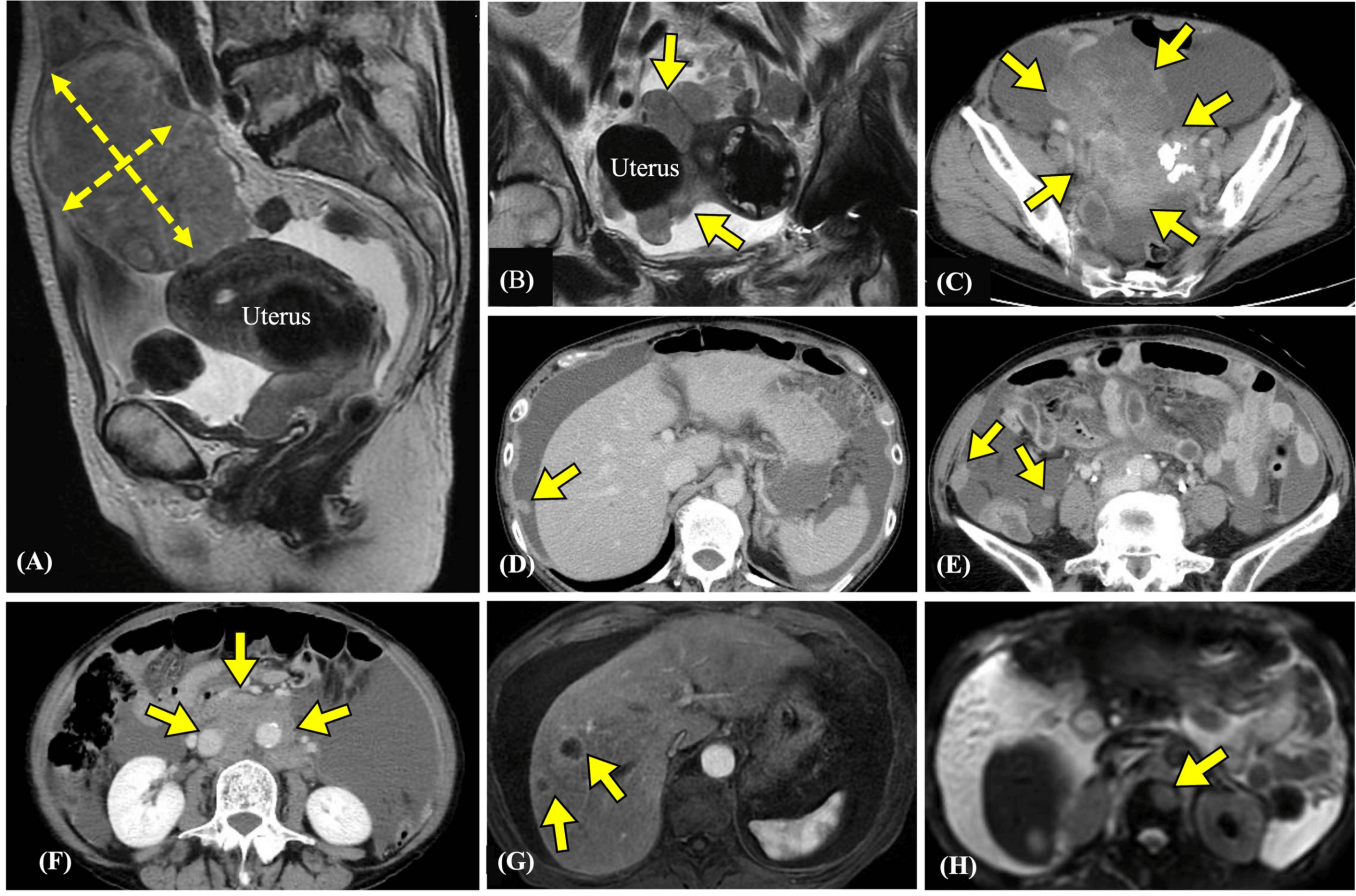
Imaging findings at initial presentation (A) MRI sagittal view showing a solid mass in the pelvis (12 × 7.8 cm). (B) MRI coronal view showing a solid mass around the uterus. (C) CT image showing a solid mass in the right adnexa and uterus. (D, E) CT image showing peritoneal dissemination. (F) CT image showing para-aortic lymphadenopathy. (G) MRI image showing multiple liver metastases. (H) MRI image showing vertebral bone metastases. CT: computed tomography, MRI: magnetic resonance imaging

A 66-year-old woman (gravida 0, para 0) presented to our hospital with a two-week history of lower abdominal distention and loss of appetite. Her history included appendicitis during childhood and a femoral fracture at the age of 64. She had a family history of malignancies. Her mother was diagnosed with breast and uterine cancer at an older age, though the exact age of onset was unknown. Additionally, a maternal aunt was diagnosed with gynecologic cancer, with the age of onset also unknown. Transvaginal ultrasound demonstrated a large solid tumor and ascites in the pelvis. Laboratory tests showed a significantly increased lactate dehydrogenase (LDH) level of 1484 U/L and a cancer antigen 125 (CA125) level of 1165 U/mL. Her serum calcium concentration was normal. Computed tomography (CT) and magnetic resonance imaging (MRI) revealed a 12-cm solid tumor with irregular margins in the right adnexa and uterus. CT additionally showed peritoneal dissemination and pelvic and para-aortic lymphadenopathy. MRI further identified multiple metastases in the liver and vertebral bones (Figure 1).

**Figure 2:**
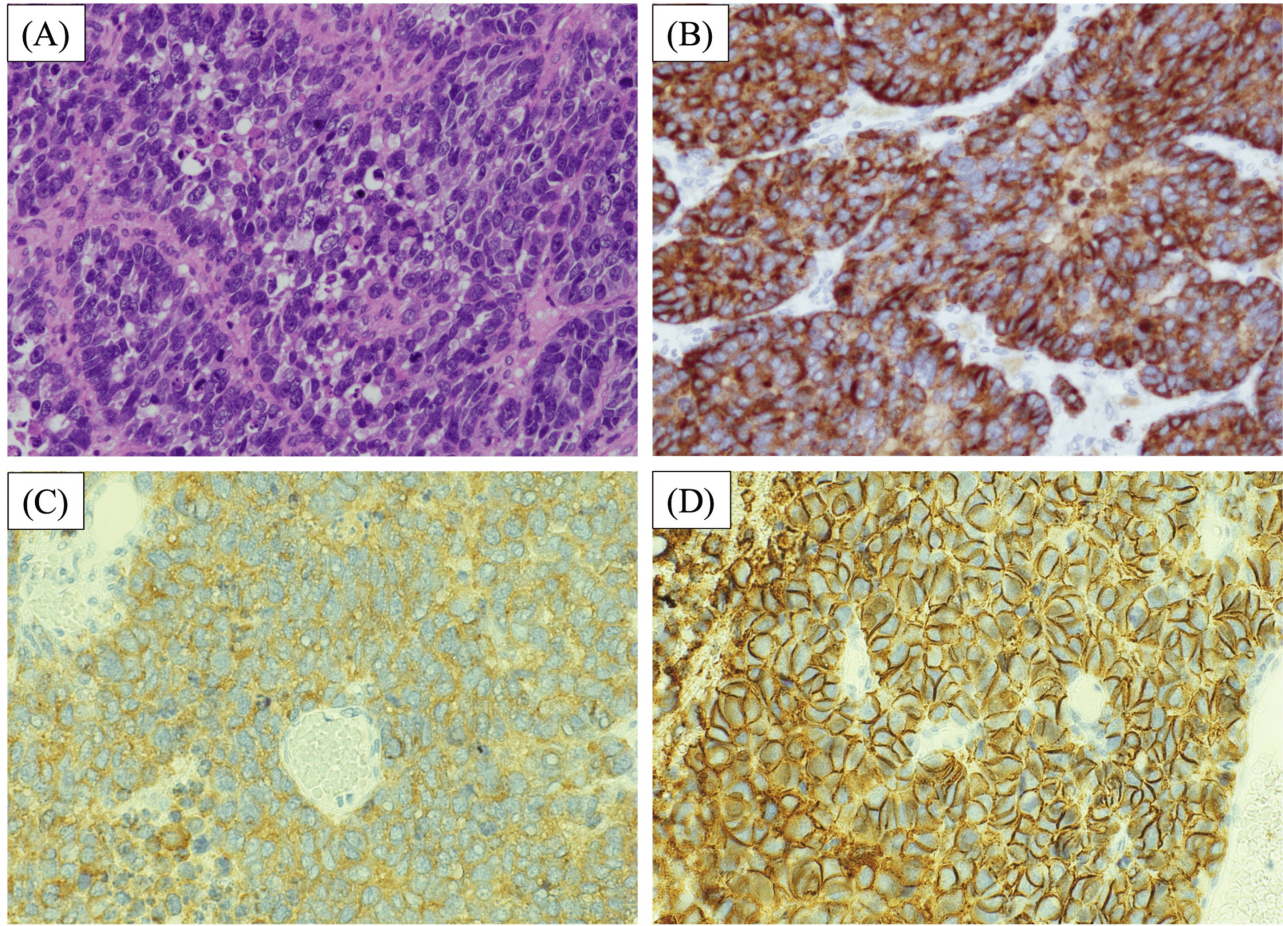
Histopathological findings (A) Hematoxylin and eosin-stained photomicrograph of the operative specimen showing small cohesive cells with scant cytoplasm, hyperchromatic nuclei, and brisk mitotic activity (magnification 20×). (B) Immunochemistry-treated photomicrograph showing chromogranin A positivity (magnification 20×). (C) Immunochemistry-treated photomicrograph showing synaptophysin positivity (magnification 20×). (D) Immunochemistry-treated photomicrograph showing CD56 positivity (magnification 20×).

There was no evidence of pulmonary nodules or masses. An ascitic cell block analysis only revealed atypical cells and did not lead to a definitive diagnosis. Therefore, the preoperative diagnosis was primary ovarian or uterine malignancy. The patient underwent a laparotomy for diagnostic and palliative purposes. During the laparotomy, a 12-cm hemorrhagic tumor with predominantly solid and necrotic areas firmly adherent to and encasing the uterine surface was identified. Peritoneal dissemination from the pelvis to the diaphragm and 3400 mL of hemorrhagic ascites were also noted. Total abdominal hysterectomy and bilateral salpingo-oophorectomy were performed. The cancer was unresectable, but no attempts were made to achieve further cytoreduction. Thus, the surgery was suboptimal. Histological examination of the operative specimen revealed a solid hemorrhagic and necrotic tumor composed of small cohesive cells with scant cytoplasm, hyperchromatic nuclei, and high mitotic activity. Immunohistochemistry demonstrated that the tumor cells were positive for chromogranin A, synaptophysin, and CD56. The above findings are consistent with neuroendocrine differentiation (Figure 2).

There were no other components typical of surface epithelial-stromal tumors. Histopathological examination revealed no mucinous, serous, endometrioid, or teratomatous elements. The final diagnosis of SCCOPT, stage IVB (T3cN1M1), was based on histological findings, the immunohistochemical staining pattern, and clinical findings.

**Figure 3:**
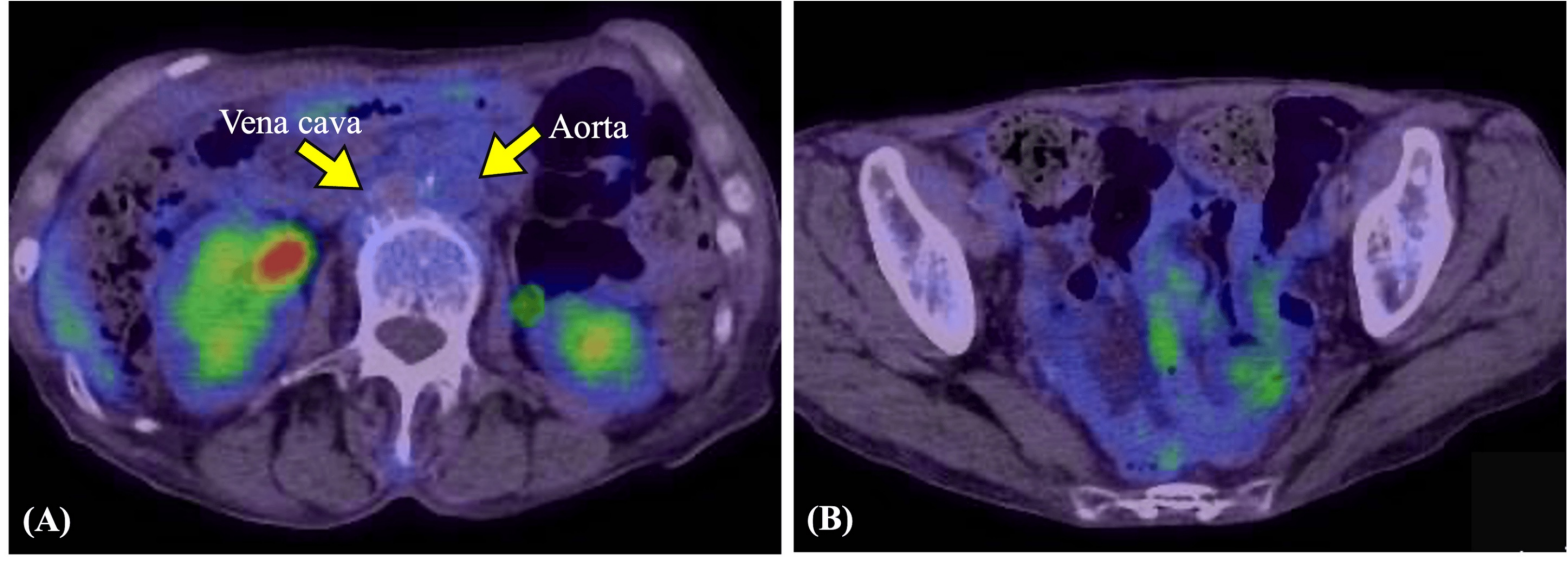
Imaging findings after the completion of chemotherapy PET image showing an absence of abnormal fluorodeoxyglucose-avid lesions. (A) PET image showing no fluorodeoxyglucose-avid lesions in the para-aortic lymph node area. (B) PET image showing no fluorodeoxyglucose-avid lesions in the pelvis. PET: positron emission tomography

The patient’s postoperative course was uneventful. However, a CT performed four weeks after surgery revealed notable disease progression, including multiple liver metastases and para-aortic lymphadenopathy. Relevant postoperative laboratory results showed an elevated LDH level of 2185 U/L, a CA 125 level of 459 U/mL, and a neuron-specific enolase (NSE) level of 468 ng/mL. She then underwent six cycles of chemotherapy with paclitaxel (175 mg/m²), carboplatin (AUC 5), and bevacizumab (15 mg/kg). After completion of this chemotherapy, a positron emission tomography scan showed no fluorodeoxyglucose-avid lesions (Figure 3).

The serum concentrations of LDH, CA125, and NSE had decreased to within normal limits after completion of this chemotherapy.

Homologous recombination deficiency testing was approved to help select maintenance therapy; this and identifying a tumor *BRCA* mutation was positive. A g*BRCA* gene mutation test was performed, and it identified the pathogenic variant *BRCA2* c.4587dupG, which causes a translational frameshift and premature termination of *BRCA2* protein synthesis. Subsequently, genetic counseling was provided to the patient. The patient had an older sister, a younger sister, and several nephews and nieces, all of whom resided far away and were not present during the genetic counseling session. These family members did not express interest in receiving genetic counseling and did not undergo genetic testing. Additionally, no further genetic testing, such as gene panel testing, was performed on the patient.

**Figure 4:**
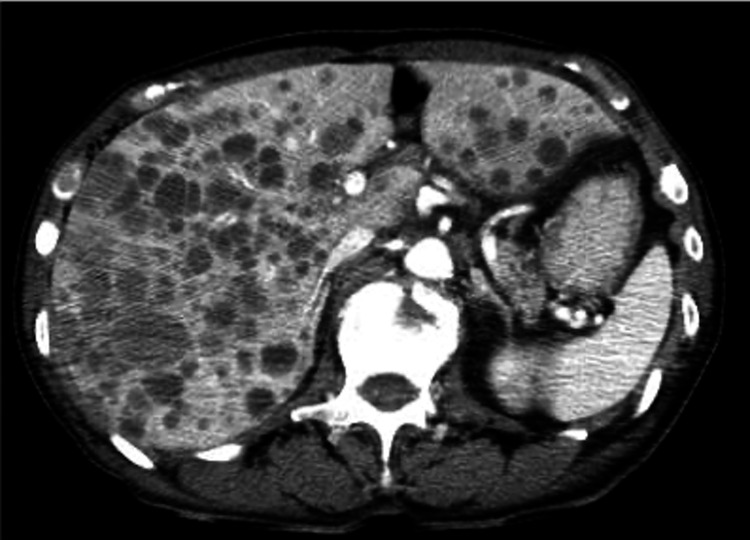
Imaging finding at disease progression CT image showing multiple liver metastases. CT: computed tomography

After completion of chemotherapy, maintenance therapy with a combination of PARPi (olaparib) and bevacizumab was planned. However, uncontrolled hypertension during this treatment prompted a switch to monotherapy with niraparib, an alternative PARPi. Five months after the completion of chemotherapy and four months after initiating maintenance therapy, a CT scan revealed no evidence of disease progression. Laboratory tests showed a slightly elevated NSE level of 37.2 ng/mL, while the CA125 level was 14.6 U/mL, remaining within the normal limit. Only one month after the CT scan, however, the patient reported nausea and a decreased appetite. Laboratory testing showed a major increase in the LDH level to 5428 U/mL, aspartate aminotransferase level of 123 U/L, and alanine aminotransferase level of 190 U/L, NSE level of 1050 ng/mL, while the CA125 level remained within the normal limit at 15.1 U/mL. A repeat CT scan revealed multiple liver metastases (Figure 4).

Because the severe progressive hepatic dysfunction rendered the patient intolerant to any chemotherapy, the best supportive care was substituted. She died 12 months after surgery and two weeks after the diagnosis of recurrence.

## Discussion

Primary small cell carcinoma of the ovary is a rare and highly aggressive malignant neoplasm that is classified into two distinct subtypes: SCCOPT and SCCOHT [1,2].

SCCOPT most often occurs in women aged 50-60 years [1]. The histopathological and immunohistochemical features of SCCOPT are similar to those of small cell carcinoma of the lung, including the presence of small cells with hyperchromatic nuclei, scant cytoplasm, and abundant mitotic activity [6]. Tumor cells in SCCOPT are generally positive for immunohistochemical neuroendocrine markers such as chromogranin A, synaptophysin, and CD56, demonstrating that SCCOPT represents a true neuroendocrine tumor [3]. Approximately half of SCCOPT cases consist solely of small cell carcinoma, while the remainder are associated with other components. SCCOPT has been reported to co-exist with other ovarian neoplasms, including Brenner tumors, mucinous neoplasms, mature cystic teratomas, and endometrioid adenocarcinomas [7].

In contrast, SCCOHT predominantly occurs in younger women, with a mean age of 28 years [1]. Approximately 70% of SCCOHT patients present with paraneoplastic hypercalcemia [1]. Histopathologically, SCCOHT demonstrates morphological diversity [3], characterized by small cells often arranged in follicle-like spaces, with larger cells exhibiting rhabdoid morphology in about 50% of cases [3]. The most distinctive feature of SCCOHT is the loss of nuclear expression of *SMARCA4*/BRG1 in over 90% of cases [3]. Unlike SCCOPT, SCCOHT tumors are generally negative for immunohistochemical neuroendocrine markers [3].

Primary small cell carcinoma of the ovary accounts for fewer than 1% of ovarian cancers [1]. Among the two subtypes, SCCOPT is rarer than SCCOHT [2]. The largest review of SCCOPT available in the literature includes only 38 patients [4], while the most comprehensive review of SCCOHT includes 306 patients [8].

Although there are no specific treatment guidelines for SCCOPT due to its rarity, the standard treatment combines surgery and chemotherapy. However, the prognosis is very poor even when diagnosed at an early stage, with a median survival of approximately 24 months [4].

Surgical treatment is similar to common ovarian epithelial cancers, which can usually only be differentiated from SCCOPT by their pathological features [1]. In operable cases, standard primary surgical debulking is the treatment of choice [1]. However, for selected patients with bulky disease who seem to be unsuitable for primary debulking surgery, neoadjuvant chemotherapy followed by interval debulking surgery is considered [1]. Interestingly, Münstedt K et al. demonstrated that the success of surgery, as indicated by R0/R1 resection, has not been a significant prognostic factor in this rare malignancy [4]. This finding contrasts with the established prognostic importance of optimal cytoreduction in epithelial ovarian cancers, suggesting a distinct biological behavior of SCCOPT.

Optimal chemotherapy regimens have not been established because of the rarity of this condition. In general, chemotherapeutic regimens for SCCOPT primarily involve platinum-doublet chemotherapy, which usually comprises a platinum-based agent in combination with etoposide, as used for small cell lung cancer [1]. Combinations, including paclitaxel, alkylating agents, or anthracyclines, used for epithelial ovarian cancer, may also serve as components of these regimens [3,4,6,7,9]. Eichhorn et al. have described the use of more intensive chemotherapy regimens, such as combinations of cyclophosphamide, cisplatin, doxorubicin, etoposide, and vincristine; however, the efficacy and potential benefits of these more intensive chemotherapy protocols have not been conclusively established [10].

In common ovarian epithelial cancer, the efficacy of maintenance therapy with PARPi has been well-established by g*BRCA* status [5]. However, for SCCOPT, there is a paucity of data regarding maintenance therapy, and its efficacy remains poorly understood.

Genomic mutations in SCCOPT are poorly characterized due to their rarity. Yaghmour et al. analyzed the data of 46 patients with neuroendocrine cancers of the ovary, including 10 with small cell carcinoma of the ovary, and found that actionable mutations were rare; only one of the patients with small cell carcinoma of the ovary had a somatic *BRCA2* mutation. They did not examine any g*BRCA* mutations in this report [11]. Similarly, in a case series of 12 neuroendocrine cancers of the ovary by Xing et al., one *BRCA*-positive case was identified in a large cell neuroendocrine carcinoma but not in small cell carcinomas of the ovary [12]. While a g*BRCA* mutation has been reported in the other type of small cell carcinoma of the ovary, SCCOHT [13], there has been no evidence to suggest an association between *BRCA* mutations and SCCOPT, as these are considered distinct entities. To our knowledge, our patient represents the first reported with SCCOPT and a g*BRCA* mutation. Notably, histopathological examination of our patient’s tumor revealed it to be composed exclusively of SCCOPT, without evidence of other ovarian tumor components. This finding suggests a potential association between the g*BRCA* mutation and the SCCOPT component in this patient, a correlation not previously reported in the literature.

Our patient had a g*BRCA2* c.4587dupG pathogenic mutation, causing a translational frameshift with premature termination of *BRCA2* protein synthesis. This mutation is located within the ovarian cancer cluster region of *BRCA2*, which is where half of Japanese patients with ovarian cancer and g*BRCA2* mutations were reported to have variants [14]. This mutation was also documented in an individual with a family history of pancreatic cancer [15].

Previous reports have documented the use of paclitaxel and carboplatin (TC) chemotherapy in four patients (Table 1) [3,6,9].

**Table 1:** Case reports and clinicopathological characteristics of SCCOPT treated with TC chemotherapy BSO: bilateral salpingo-oophorectomy, IDS: interval debulking surgery, LTF: lost to follow-up, OMT: omentectomy, PALA: para-aortic lymphadenectomy, PLA: pelvic lymphadenectomy, TAH: total abdominal hysterectomy, TC: paclitaxel and carboplatin, SCCOPT: small cell carcinoma of the ovary, pulmonary type

	Age (years)	FIGO stage	Surgical treatment	Chemotherapy	Recurrence (months)	Overall survival (months)
							Case 1: Gupta et al. (2021) [3]	60	IV	IDS (remove ovarian masses)	6 cycles of TC before surgery	1	LTF
							Case 2: Gupta et al. (2021) [3]	60	IV	IDS (BSO + OMT)	6 cycles of TC before surgery	+ unknown interval	≧30
Kurasaki et al. (2013) [6]	54	IIIA	TAH + BSO + OMT	6 cycles of TC after surgery	-	≧22
Suzuki et al. (2007) [9]	49	IC	TAH + BSO + OMT + PLA + PALA	6 cycles of TC after surgery	-	≧36
Current case	66	IV	TAH + BSO	6 cycles of TC + bevacizumab after surgery, niraparib maintenance therapy	11	12

The tumors of all four patients consisted exclusively of SCCOPT, with no coexisting components, as seen in our patient. Two of the four patients were diagnosed with stage IV disease and underwent neoadjuvant chemotherapy followed by interval debulking surgery. One of these had a recurrence one month after the completion of the combination therapy and was lost to follow-up. The other had a recurrence after an unknown interval and was alive with the disease at 30 months [3]. The two other patients were diagnosed with stage IIIA and stage IC, underwent surgery followed by chemotherapy, and were alive and recurrence-free at 22 and 36 months, respectively [6,9].

Our patient underwent surgery followed by chemotherapy, and subsequently, maintenance therapy with PARPi was initiated. To our knowledge, this is the first reported case of SCCOPT treated with PARPi maintenance therapy. Our patient’s initial good response to platinum-based chemotherapy, coupled with the presence of the g*BRCA* mutation, raised our expectations for a favorable outcome with PARPi maintenance therapy. However, she relapsed rapidly with multiple liver metastases only five months after starting maintenance therapy and died 12 months after surgery.

Previous reviews have reported that SCCOPT has a poor prognosis regardless of stage or extent of surgical resection, with a median survival of approximately 24 months across all stages [4]. Our patient demonstrated a worse outcome than the reported prognosis. Moreover, the clinical course in our patient was not superior to previously reported cases treated with TC chemotherapy, suggesting that PARPi maintenance therapy may have limited efficacy against SCCOPT, even in the presence of g*BRCA* mutations.

Numerous mechanisms of acquired resistance to PARPi have been described [16]. A primary mechanism involves the restoration of homologous recombination repair. This restoration can occur through various pathways, including secondary *BRCA* mutations, epigenetic alterations, or mutations in specific functional proteins involved in homologous recombination repair. Additionally, mechanisms independent of homologous recombination repair restoration have been identified. These include increased drug efflux, which reduces intracellular concentrations of PARPi, and PARP1 mutations that prevent PARPi trapping on DNA, thereby diminishing their efficacy. However, it is important to note that no established indicators or laboratory tests can definitively identify PARPi resistance. In our patient, the specific factors contributing to the development of resistance remain unknown.

Despite multimodal treatment approaches combining surgery and chemotherapy, the prognosis for SCCOPT remains poor. Moreover, unlike epithelial ovarian cancer, maintenance therapy has not yet been established for SCCOPT. Therefore, developing effective maintenance therapy is a critical unmet need. Further research is necessary to investigate the role of PARPi in the maintenance therapy of SCCOPT, particularly in the context of g*BRCA* mutations.

## Conclusions

To our knowledge, this is the first report of a patient with SCCOPT and a g*BRCA* mutation receiving PARPi maintenance therapy. Despite the g*BRCA* mutation and favorable response to platinum-based chemotherapy, the patient’s tumor was resistant to a PARPi, resulting in early recurrence and death within 12 months of surgery. In our patient, maintenance therapy appears to have had a minimal impact on treating SCCOPT, with the outcome not being superior to that of previously reported patients. Further research is necessary to determine the role of PARPi and establish more effective treatment strategies for SCCOPT.
